# Persistence of Toscana virus in sugar and blood meals of phlebotomine sand flies: epidemiological and experimental consequences

**DOI:** 10.1038/s41598-023-32431-9

**Published:** 2023-04-05

**Authors:** Lison Laroche, Nazli Ayhan, Rémi Charrel, Anne-Laure Bañuls, Jorian Prudhomme

**Affiliations:** 1 UMR MIVEGEC, Université de Montpellier – IRD 224 – CNRS 5290, 911 Avenue Agropolis, 34394 Montpellier, France; 2 UVE, Aix Marseille Université – IRD 190 – Inserm 1207 – AP-HM Hôpitaux Universitaires de Marseille, Marseille, France; 3 INTHERES, Université de Toulouse, INRAE, ENVT, Toulouse, France

**Keywords:** Entomology, Viral infection, Viral vectors, Virus-host interactions

## Abstract

Many virological studies have tested the persistence of enveloped RNA viruses in various environmental and laboratory conditions and shown their short-term persistence. In this article, we analyzed Toscana virus (TOSV) infectivity, a pathogenic sandfly-borne phlebovirus, in two different conditions: in the sugar meal and blood meal of sand flies. Our results showed that TOSV RNA was detectable up to 15 days in sugar solution at 26 °C and up to 6 h in blood at 37 °C. Moreover, TOSV remains infective for 7 days in sugar solution and for minimum 6 h in rabbit blood. TOSV has shown persistent infectivity/viability under different conditions, which may have important epidemiological consequences. These results strengthen new hypotheses about the TOSV natural cycle, such as the possibility of horizontal transmission between sand flies through infected sugar meal.

## Introduction

Toscana virus (TOSV) is an enveloped, protein encapsidated, tri‐segmented RNA virus that belongs to the *Phlebovirus* genus (family *Phenuiviridae,* order Bunyavirales)^1^. It is an arthropod-borne virus transmitted to humans through the bite of infected female sand flies^2^. The geographical distribution of TOSV is highly dependent on this vector abundance^1^. Human cases are observed during the warm season, with a peak during summer related to a high vector activity^3^. TOSV infections are endemic in the Mediterranean basin and considered frequent during the warm season even though they remain neglected^4^. Most infections are believed to be asymptomatic or mildly symptomatic even though the exact proportion remains unknown^5^. TOSV has a particular neurotropism and is a major cause of meningitis and encephalitis in endemic areas^3^. Few symptomatic neuroinvasive cases have been reported, with an estimated incubation period of 12.1 days^6^.

Until now, little is known about the natural cycle of TOSV and its transmission routes. Since there is no concrete evidence that vertebrate host species are reservoirs, it has been hypothesized that vector sand fly species can be the primary reservoir of TOSV^1,7^. Transovarial and venereal transmissions have been observed experimentally with a low infection rate in *Phlebotomus perniciosus*, the main identified vector of TOSV throughout the Mediterranean basin^3,8^. These transmission pathways are probably not efficient enough to maintain TOSV in the wild. Alternative transmission routes should exist although they remain unknown. For example, in aphids, the horizontal transmission of insect densoviruses was demonstrated through honeydew sugar meals^9^.

As both male and female sand flies feed mainly on plant nectar and honeydew^10^, it is important to explore the possibility of TOSV transmission between individuals during sugar meals. Only one study using Massilia virus, another phlebovirus genetically close to TOSV, has evidenced the possible transmission through this pathway^7^. Enveloped and RNA genome viruses have limited stability in the environment outside the host^11,12^. However, it is essential to determine arbovirus persistence/viability in different conditions to better understand the strategies employed by arboviruses to persist in the natural environment^13^. In this study, we assessed TOSV stability and infectivity in sugar meals and in blood meals, sequentially, to estimate epidemiological consequences.

## Results

### TOSV persistence in blood

**Figure 1:**
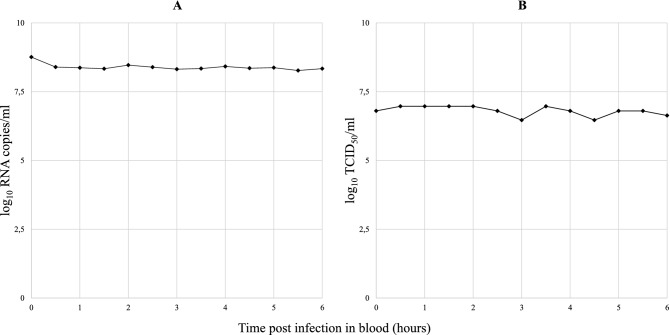
(**A**) Toscana virus RNA load (log_10_ copies/ml) and (**B**) TOSV infectivity (TCID_50_/ml) in blood over time.

After six hours at 37 °C in heat-inactivated rabbit blood, the quantity of TOSV ranged from 8.3 to 8.8 log_10_ RNA copies/ml (Fig. 1A). There was no decrease in the quantity of virus RNA in blood over time. TOSV infectivity decreased less than 0.5 log over time after infection (Fig. 1B). All samples were positive for cytopathic effects. TOSV remains infectious for at least six hours in blood at 37 °C.

### Toscana virus persistence in sugar

**Figure 2:**
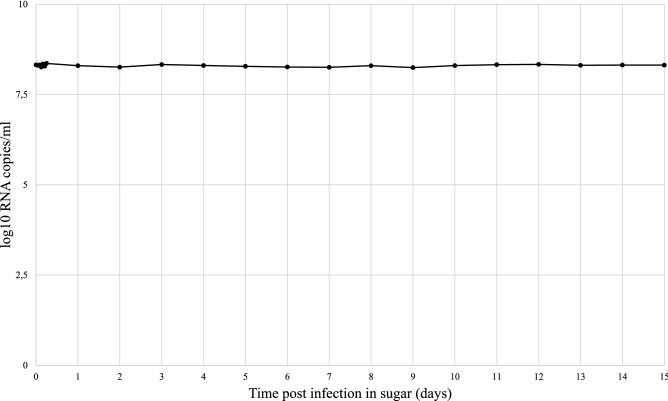
Toscana virus RNA load (log_10_ copies/ml) in sugar solution over time post infection.

TOSV quantity ranged from 8.2 to 8.4 log_10_ RNA copies/ml in sugar solution over the first six hours and up to 15 days post infection (Fig. 2). There was no decrease in the quantity of virus genome in the sugar solution over 15 days.

**Figure 3:**
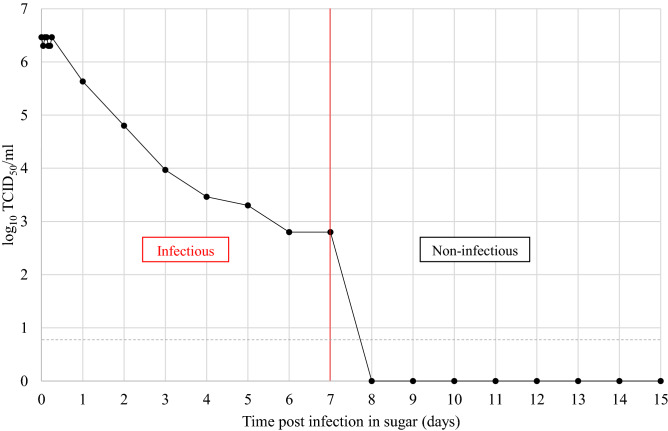
Toscana virus infectivity (TCID_50_/ml) in the sugar solution over time post infection. The red vertical line represents the boundary between infectious and non-infectious samples. The grey dashed horizontal line corresponds to the limit of detection of infectivity (0.8 log_10_ TCID_50_/ml).

The infectivity experiments showed that TOSV titration ranged from 6.3 to 6.5 log10 TCID_50_/ml during the first six hours and began to decrease one log per day until day seven post infection (Fig. 3). TOSV remains infectious during seven days post infection.

## Discussion

The persistence time of a pathogen in the environment can strongly affect disease dynamics^14^. Several studies on the persistence of arboviruses in different systems (humans, animals, vector insects), in vitro (cell line cultures) or according to environmental conditions (e.g., temperature, pH, light) provided additional information on the virus natural cycle^15–21^ (22–28). A recent study demonstrated the persistence of the Chikungunya virus in liquid environments (packed red blood cells, plasma, and CHIKV-spiked water) in the dark^20^. Pezzi et al.^20^ provided new insights into the main factors responsible for the stability of enveloped RNA viruses outside their hosts.

In this study, we first explored TOSV persistence in rabbit blood, which is essential information for standardized experimental infection studies. The aim was to check whether sand flies took the same amount of virus at the beginning than at the end of the blood feeding. Viral RNA was detectable at a stable amount for up to six hours in blood at 37 °C and TOSV remained infective throughout this period. Thus, sand flies feeding at the end of the blood feeding took the same quantity of virus as those feeding at the beginning. This justifies the exposure to blood for six hours as is the case in standard sand fly colony maintenance^22^. This result was essential to implement experimental infection protocols, as most sand flies start to take their blood meal within a few hours after the beginning of the exposure^22^. This is not the case for all hematophagous insects reared in laboratories. For example, for *Aedes* or *Anopheles* mosquitoes, an artificial blood feeding of 20–30 min is sufficient to obtain 90% of engorged females^23^. Since the blood may clot over time, it is difficult to estimate whether TOSV infectivity remains stable after six hours^24^. It is however essential to determine virus persistence in human blood as well to estimate the transfusion contamination risks. The risk of TOSV transmission to virus-naive persons by blood transfusions and organ transplant is suspected but still needs to be assessed^25^. The characteristics required for transmission by transfusion have been described for many infectious disease pathogens, including virus presence in the blood of asymptomatic donors and virus persistence in blood during storage^26^. For example, blood transfusion is a likely route of Zika virus transmission as its RNA has been shown to persist in the red blood cells of asymptomatic blood donors for several months^27,28^. Arbovirus RNA can be present in human saliva, semen, urine and faeces, as in the case of the Zika virus^12^, and TOSV which was found in urine and semen^29,30^. Other possible means of transmission of TOSV need to be explored.

In general, viruses can be excreted from infected hosts into the environment. Once viruses are outside their cellular hosts and in the environment, they have the potential to persist in order to reach other hosts again^31^. Thus, we sought to evaluate the persistence of TOSV infectivity in sugar meals, which are considered as the main energy source of sand flies. We show that the TOSV genome remains stable in sugar solution: viral RNA was detectable at a constant level for up to 15 days. In addition, the infection stability assay confirmed that TOSV infectivity lasts up to seven days. The assay also suggests that TOSV could be transmitted from infected to uninfected sand flies during sugar meals. An experimental study showed that another *Phlebovirus* (Massilia virus) could be transmitted between sand flies either by co-feeding or by an infected sugar source such as plant sap^7^. By investigating the transmission route of phleboviruses, they showed that both sand fly genders became infected through the sugar meal containing virus. However, in this study, only RNA viral loads were measured and not the titers in TCID_50,_ which are necessary for tittering infectious virus. Other arboviruses of the *Flaviviridae* family have been shown to be expectorated by infected mosquitoes during the sugar feeding process^32^. The indirect transmission of pathogens through another source of sweet meals (e.g., fruit) has been demonstrated for the Marburg and Ebola viruses between fruit-eating mammalian hosts. Studies showed that these infectious viruses could persist up to 6 h on different fruits^33,34^. Consequently, the hypothesis of TOSV horizontal transmission between sand flies via a sugar meal remains to be explored, as this transmission pathway could play an important role in the natural cycle of *Phlebovirus*. Furthermore, it would be necessary to test TOSV infectivity/viability under different temperatures and UV-light exposures, as they might be important factors under natural conditions. Finally, the transmission of TOSV via a sugar solution could represent an alternative for laboratories unable to carry out experimental infections with infected blood.

In conclusion, this study assessed TOSV infectivity in two environments, sugar and blood. The amount of viral RNA remained constant over a long period of time and no viral replication occurred. Although TOSV is an enveloped RNA virus, known for its limited stability in the environment outside the host, our study clearly demonstrates its persistence in different environments. In general, long-term viral persistence increases indirect transmission risks for hosts or arthropod vectors^35^.

## Methods

### Ethical statement

Rabbit blood draws performed in the context of this study were approved by the Animal Care and Use Committee named “Comité d’Ethique pour l’Expérimentation Animale Languedoc Roussillon n°36” under protocol number 2018022712203932. Rabbits, coming from the animal facility at the French National Research Institute for Sustainable Development, were not subjected to anesthesia, analgesia or sacrifice. The chicks come from the Experimental Infectiology Platform of Nouzilly (INRAE Centre Val de Loire, France). They were killed according to the Directive 2010/63/EU (Appendix IV) appropriate to the species. The chicks were dead before the use of tissue samples for experimental infection, in accordance with article R214-89 of the French Code rural et de la pêche maritime, Section 6. This study is reported in accordance with ARRIVE guidelines. We declare that all methods were performed in accordance with the relevant guidelines and regulations.

### Preparation and storage of TOSV aliquot

Lyophilised TOSV aliquots (strain MRS2010, lineage B) were provided by the UVE laboratory (Unité des Virus Emergents, Marseille, France). Vero E6 (African green monkey kidney) cells were grown in monolayers in Minimum Essential Medium (MEM, GIBCO) complemented with 7% heat-inactivated Fetal Bovine Serum (FBS, EUROBIO SCIENTIFIC), 1% l-glutamine (GIBCO) and 1% penicillin–streptomycin (GIBCO) at 37 °C with 5% CO2. Stock of TOSV was obtained by dissolving the lyophilisates in sterilized pure water. Vero E6 cells were infected with 0.1 multiplicity of infection (MOI) and supernatant media were harvested on day 5 post infection. TOSV stocks at a concentration of 4.2 × 106 50% tissue culture infective doses (TCID_50_/ml) were aliquoted in 2 ml cryotubes and stored at − 80 °C.

### TOSV persistence in blood

**Figure 4:**
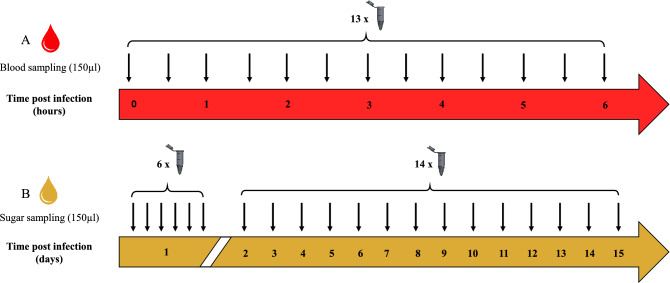
Sampling method for the assessment of TOSV persistence (**A**) in rabbit blood at 37 °C and (**B**) in sugar solution at 26 °C.

The experiment was performed under the standard conditions for sand fly experimental infections (i.e., 26 °C, 80% relative humidity and 1% luminosity)^36^. A glass feeder, covered with chicken skin, was filled with heat-inactivated rabbit blood infected with 10^6^ TCID_50_/ml of TOSV. The glass feeder was connected to a water bath with external circulation to maintain blood at a constant temperature of 37 °C. As in standard experimental infections, this system was maintained during six hours^22^. In order to quantify TOSV genome stability and infectivity kinetics, 150 µl of infected blood was collected every 30 min (after blood homogenization) (Fig. 4A) and stored in cryotubes at − 80 °C for subsequent titration.

### TOSV persistence in sugar solution

The experiment was performed by infecting a sugar solution with 10^6^ TCID_50_/ml of TOSV, according to the standard conditions for sand fly experimental infections, during 15 days. The sugar solution was the same as the one used for the maintenance of sand fly colonies: 50% organic brown sugar in sterile distilled water prepared as described previously^37,38^. The infected sugar solution was collected (150 µl) every hour during six hours on the first day and once a day until day 15 (Fig. 4B) in order to quantify TOSV genome stability and infectivity kinetics. The solution was stored in cryotubes at − 80 °C for subsequent titration.

### Kinetics of TOSV genome stability

The viral loads of blood and sugar samples were analyzed by RT-qPCR. A total of 200 μl was used for nucleic acid extraction with the QIAcube (QIAGEN) machine and the Virus Extraction Mini Kit (QIAGEN). The RT-qPCR assays were performed with a SuperScript III Platinum One-Step RT-qPCR Kit with ROX (Invitrogen—THERMO FISHER SCIENTIFIC) on a QuantStudio 12 K Flex thermocycler (THERMOFISHER). A volume of 5 µl of RNA was added to 20 µl of mix containing 12.5 µl of 2X Reaction Mix, 0.5 µl of Superscript III RT/Platinum Taq Mix, and 10 µM of STOS primers and probes^39^. Negative (pure water) and positive controls (at 4.81 × 10^4^ RNA in vitro transcribed copies/µl as described by Beckert and Masquida^40^) were included in each RT-qPCR run. Samples with a Ct value < 40 were considered positive.

### Kinetics of TOSV infectivity

RT-qPCR can be used as a surrogate to measure viral RNA copies. However, the results need to be correlated to TCID_50_ data which are the only measures that inform on the capacity of transmission and infection of the virus. TOSV titers in blood and sugar samples were determined by end-point dilution assay. Tenfold dilutions were used to infect confluent Vero E6 cells in a 96-wells plate in MEM (5% FBS, 1% penicillin–streptomycin, 1% l-Glutamine, 3% amphotericin (GIBCO) at 37 °C in 5% CO_2_. Wells were classified as positive (cytopathic effect) *versus* negative (no cytopathic effect) at five days post infection. TCID_50_/ml was calculated according to Reed and Muench^41^.

## Data Availability

All resources used in this article are provided and all the analyses are detailed allowing the assessment or verification of the manuscript’s findings.

## References

[1] Ayhan N, Prudhomme J, Laroche L, Banuls AL, Charrel RN (2020). Broader geographical distribution of Toscana virus in the Mediterranean region suggests the existence of larger varieties of sand fly vectors. Microorganisms.

[2] Depaquit J, Grandadam M, Fouque F, Andry PE, Peyrefitte C (2010). Arthropod-borne viruses transmitted by *Phlebotomine sandflies* in Europe: A review. Euro Surveill.

[3] Charrel RN (2005). Emergence of Toscana virus in Europe. Emerg. Infect. Dis..

[4] Charrel RN, Bichaud L, de Lamballerie X (2012). Emergence of Toscana virus in the mediterranean area. World J. Virol..

[5] Braito A (1997). Evidence of Toscana virus infections without central nervous system involvement: a serological study. Eur. J. Epidemiol..

[6] Laroche L (2021). Incubation period for neuroinvasive Toscana virus infections. Emerg. Infect. Dis..

[7] Jancarova M (2019). Experimental infection of sand flies by Massilia virus and viral transmission by co-feeding on sugar meal. Viruses.

[8] Tesh RB, Modi GB (1987). Maintenance of Toscana virus in *Phlebotomus perniciosus* by vertical transmission. Am. J. Trop. Med. Hyg..

[9] Van Munster M (2003). Characterization of a new densovirus infecting the green peach aphid *Myzus persicae*. J. Invertebr. Pathol..

[10] Cameron M, Pessoa F, Vasconcelos A, Ward R (1995). Sugar meal sources for the phlebotomine sandfly *Lutzomyia longipalpis* in Ceara State, Brazil. Med. Vet. Entomol..

[11] McKnight KL, Lemon SM (2017). Ins and outs of picornaviruses. Nature.

[12] Muirhead A (2020). Zika virus RNA persistence in sewage. Environ. Sci. Technol. Lett..

[13] Mlera L, Melik W, Bloom ME (2014). The role of viral persistence in flavivirus biology. Pathog. Dis..

[14] Lequime S, Paul RE, Lambrechts L (2016). Determinants of arbovirus vertical transmission in mosquitoes. PLoS Pathog..

[15] Kuno G (2001). Persistence of arboviruses and antiviral antibodies in vertebrate hosts: its occurrence and impacts. Rev. Med. Virol..

[16] Nemeth N (2009). Persistent West Nile virus infection in the house sparrow (*Passer domesticus*). Arch. Virol..

[17] Lequime S, Lambrechts L (2014). Vertical transmission of arboviruses in mosquitoes: A historical perspective. Infect. Genet. Evol..

[18] Nuttall PA, Jones LD, Labuda M, Kaufman WR (1994). Adaptations of arboviruses to ticks. J. Med. Entomol..

[19] Way SJ, Lidbury BA, Banyer JL (2002). Persistent Ross River virus infection of murine macrophages: an in vitro model for the study of viral relapse and immune modulation during long-term infection. Virology.

[20] Pezzi L (2021). Long-term infectivity of Chikungunya virus stored in the Dark at 4 °C. Vector Borne Zoonotic Dis..

[21] Carvalho CA, Silva JL, Oliveira AC, Gomes AM (2017). On the entry of an emerging arbovirus into host cells: Mayaro virus takes the highway to the cytoplasm through fusion with early endosomes and caveolae-derived vesicles. PeerJ.

[22] Prudhomme, J. *et al.* Establishment and maintenance of two sand fly colonies (*Phlebotomus perniciosus* and *Phlebotomus papatasi*) for experimental infections. *J. Med. Entomol.* (2022).

[23] Novak M, Berry W, Rowley W (1991). Comparison of four membranes for artificially bloodfeeding mosquitoes. J. Am. Mosq. Control Assoc..

[24] Brake MA (2019). Assessing blood clotting and coagulation factors in mice. Curr. Protoc. Mouse Biol..

[25] Brisbarre N (2011). Seroprevalence of Toscana virus in blood donors, France, 2007. Emerg. Infect. Dis..

[26] Stramer S (2014). Current perspectives in transfusion-transmitted infectious diseases: Emerging and re-emerging infections. ISBT Sci. Ser..

[27] Stone M (2020). Zika virus RNA and IgM persistence in blood compartments and body fluids: A prospective observational study. Lancet Infect. Dis..

[28] Musso D (2014). Potential for Zika virus transmission through blood transfusion demonstrated during an outbreak in French Polynesia, November 2013 to February 2014. Euro. Surveill..

[29] Matusali G (2022). Infectious Toscana virus in seminal fluid of young man returning from Elba island, Italy. Emerg. Infect. Dis..

[30] Ergunay K (2015). Urinary detection of toscana virus nucleic acids in neuroinvasive infections. J. Clin. Virol..

[31] Sobsey MD, Meschke JS (2003). Virus Survival in the Environment with Special Attention to Survival in Sewage Droplets and Other Environmental Media of Fecal or Respiratory Origin.

[32] Hurk AFVD (2007). Expectoration of flaviviruses during sugar feeding by mosquitoes (Diptera: Culicidae). J. Med. Entomol..

[33] Amman BR, Schuh AJ, Albariño CG, Towner JS (2021). Marburg virus persistence on fruit as a plausible route of bat to primate filovirus transmission. Viruses.

[34] Leroy E, Gonzalez J, Pourrut X (2007). Ebolavirus and other filoviruses: Wildlife and emerging zoonotic diseases: The biology, circumstances and consequences of cross-species transmission. Curr. Top. Microbiol. Immunol..

[35] Pirtle E, Beran G (1991). Virus survival in the environment. Rev. Sci. Tech..

[36] Vaselek S (2020). Comparative study of promastigote- and amastigote-initiated infection of *Leishmania infantum* (Kinetoplastida: Trypanosomatidae) in *Phlebotomus perniciosus* (Diptera: Psychodidae) conducted in different biosafety level laboratories. J. Med. Entomol..

[37] Volf P, Volfova V (2011). Establishment and maintenance of sand fly colonies. J. Vector Ecol..

[38] Lawyer P, Killick-Kendrick M, Rowland T, Rowton E, Volf P (2017). Laboratory colonization and mass rearing of phlebotomine sand flies (Diptera, Psychodidae). Parasite.

[39] Pérez-Ruiz M, Collao X, Navarro-Marí J-M, Tenorio A (2007). Reversetranscription, real-time PCR assay for detection of Toscana virus. J. Clin. Virol..

[40] Beckert B, Masquida B (2011). Synthesis of RNA by in vitro transcription BT-RNA: Methods and protocols. Methods Mol. Biol..

[41] Reed LJ, Muench H (1938). A simple method of estimating fifty per cent endpoints. Am. J. Epidemiol..

